# Acute Kidney Injury in Elderly Patients With Coronavirus Infectious Disease: A Study of Incidence, Risk Factors, and Prognosis in Brazil

**DOI:** 10.3389/fneph.2022.896891

**Published:** 2022-05-09

**Authors:** Bruna Kaori Yuasa, Luis Eduardo Magalhães, Paula Gabriela Sousa de Oliveira, Lais Gabriela Yokota, Pedro Andriolo Cardoso, Welder Zamoner, André Luis Balbi, Daniela Ponce

**Affiliations:** ^1^Botucatu School of Medicine, São Paulo State University, Botucatu, Brazil; ^2^Clinical Medicine Department, Botucatu School of Medicine, São Paulo State University, Botucatu, Brazil

**Keywords:** COVID-19, AKI, elderly, mortality, risk factors

## Abstract

**Introduction:**

Elderly patients with COVID-19 are at a higher risk of severity and death as not only several comorbidities but also aging itself has been considered a relevant risk factor. Acute kidney injury (AKI), one of the worst complications of SARS-CoV-2 infection, is associated with worse outcomes. Studies on AKI with COVID-19 in Latin-American patients of older age remain scarce.

**Objectives:**

To determine AKI incidence and the risk factors associated with its development, as well as to compare outcome of elderly patients with or without AKI associated with SARS-CoV-2 infection

**Methods:**

This retrospective cohort study evaluated patients with SARS-CoV2 infection admitted to a Public Tertiary Referral Hospital from 03/01/2020 to 12/31/2020, from admission to resolution (hospital discharge or death). Demographic, clinical, and laboratory data were collected from patients during hospitalization. Daily kidney function assessment was performed by measuring serum creatinine and urine output. AKI was diagnosed according to KDIGO 2012 criteria.

**Results:**

Of the 347 patients with COVID-19 admitted to our hospital during the study period, 52.16% were elderly, with a median age of 72 years (65- 80 years). In this age group, most patients were males (56.91%), hypertensive (73.48%), and required ICU care (55.25%). AKI overall incidence in the elderly was 56.9%, with higher frequency in ICU patients (p < 0.001). There was a predominance of KDIGO 3 (50.48%), and acute kidney replacement therapy (AKRT) was required by 47.57% of the patients. The risk factors associated with AKI development were higher baseline creatinine level (OR 10.54, CI 1.22 -90.61, p = 0.032) and need for mechanical ventilation (OR 9.26, CI 1.08-79.26, p = 0.042). Mortality was also more frequent among patients with AKI (46.41%vs24.7%, p < 0.0001), with death being associated with CPK level (OR 1.009, CI 1.001-1.017, p = 0.042), need for mechanical ventilation (OR 17.71, CI 1.13-277.62, p = 0.002) and KDIGO 3 (OR 2.017 CI 1.039 -3.917, p = 0.038).

**Conclusion:**

AKI was frequent among the elderly hospitalized with COVID-19 and its risk factors were higher baseline creatinine and need for mechanical ventilation. AKI was independently associated with a higher risk of death.

## Introduction

Since 11 March 2020, when officially declared, the world has been suffering from the COVID-19 pandemic (1). COVID-19, a viral disease caused by an agent of the coronavirus family called SARS-CoV-2 (2), has a very broad clinical spectrum, from asymptomatic infections or mild symptoms to severe acute respiratory distress syndrome that may lead to multi-organ failure and death (2). SARS-CoV-2 high transmissibility by aerosols (3) allowed it to spread from Wuhan, a Chinese city in Hubei province, to several countries, infecting more than 160 million people. According to the World Health Organization, more than 3 million deaths from COVID-19 were confirmed as of 18 May 2021 (4).

Among those with COVID-19 infection, elderly patients face a higher risk of severity and death as they more often present with comorbidities, such as diabetes mellitus, systemic hypertension, and cardiovascular diseases, among others (5, 6). In addition to such comorbidities, which have been associated with worse prognosis and outcomes (6), aging itself has been considered a relevant risk factor (6, 7), as advanced age is related with immunosenescence - for example, impairment of the type 1 IFN response – as well as with constant production of inflammatory mediators (7), which may increase susceptibility to a viral infection (6).

Acute kidney injury (AKI) is one of the worst complications of SARS-CoV-2 infection, and,commonly occurs in patients with respiratory failure due to COVID-19. Hirsch et al. and Zamoner et al. observed AKI in more than 75% of the patients requiring mechanical ventilation (8, 9). Others have demonstrated that AKI is associated not only with COVID-19 that has a 0.5%-29% incidence rate depending on disease severity (8–10), but also with patient prognosis, with mortality ranging from 50% to 80% (8, 10). This association among COVID-19, AKI and death might be explained by the fact that the genes encoding angiotensin-converting enzyme 2 (ACE2) and cellular transmembrane serine proteases (TMPRSS), one of the most important mediators of SARS-CoV-2 entry into host cells, are highly coexpressed in kidney podocytes and proximal tubule cells, which have been identified as candidate host cells (9, 11).

However, the pathophysiology of AKI in COVID-19 is generally accepted to be multifactorial including rhabdomyolysis with high serum levels of creatinephosphokinase; shock- associated ischemia; invasion of target cells by CD147 - that plays a role in several kidney diseases through immune-inflammatory responses and dysregulated cell cycle; microvascular changes due to endothelial cell apoptosis; thrombotic microangiopathy induced by changes in the coagulation cascade; and multi-organ dysfunction through cytokine storm (12, 13). All these mechanisms also support the development of AKI in elderly patients with COVID-19, given that aging is associated with increased serum levels of inflammatory mediators, a condition also observed in patients with severe forms of COVID-19 (6, 7).

Within this framework, elderly patients with COVID-19 can be assumed to be more likely to develop AKI, which besides being a frequent complication in in-patients infected with SARS-CoV-2, is also an extremely important predictor of worse prognosis and outcomes. Zamoner et al. have already shown that the incidence rate of AKI associated with severe COVID-19 in Brazilian patients is higher than those reported to date in their Chinese, European, and North-American counterparts (9). However, studies of AKI in Latin-American patients of older age with COVID-19 including the risk factors associated with its development and prognosis remain scarce.

Considering the above, by assessing the medical records of the patients aged above 60 years with COVID-19, who were admitted to a public tertiary hospital (both in the ICU and wards), the purpose of this study is to determine AKI incidence and the risk factors associated with its development, as well as to compare clinical outcomes, such as length of hospital stay, need for mechanical ventilation, and death, among elderly patients with or without AKI associated with SARS-CoV-2 infection.

## Methods

This observational, longitudinal, retrospective cohort study included elderly patients (defined as individuals aged 60 years and over according to the Elderly Statute and the classification of the World Health Organization, UN), who were admitted to a public tertiary hospital and diagnosed with COVID-19 from 01 March, 2020 to 31 December, 2020.

Covid-19 diagnosis was confirmed by polymerase chain reaction (PCR) test and/or serology according to the procedures standardized and validated by the São Paulo State Health Department. Data from admission to resolution (discharge or death) were obtained from patients or medical records.

At baseline, demographic and clinical data collected included age, sex, race, admission setting, presence of AKI, use of ACE inhibitor/ARBs, diuretics and nephrotoxic drugs, pulmonary disease, cardiovascular disease, liver disease, neoplasia, presence of comorbidities (such as systemic hypertension, diabetes mellitus, obesity, alcoholism, smoking, chronic kidney disease (CKD), dyslipidemia (DLP), baseline GFR and baseline creatinine.

On a daily basis, kidney function was assessed based on routine laboratory test reports for serum creatinine levels and urine output measures. AKI was diagnosed according to the KDIGO (*Kidney Disease Improving Global Outcomes*) 2012 criteria, which defines AKI as increase in serum creatinine by ≥0.3 mg/dL within 48 hours, or serum creatinine 1.5-1.9 times baseline within the last 7 days, or urine volume <0.5 mL/kg/hour for 6-12 hours.

Each patient’s daily clinical course was evaluated based on the following: hydric balance, use of nephrotoxic drugs, creatinine, urea, potassium, albumin, C-reactive protein (CRP), D-dimer, troponin, white blood cells, hemoglobin, mechanical ventilation mode, O2 arterial pressure (PaO2), fraction of inspired oxygen (FiO2), respiratory rate, positive end-expiratory pressure (PEEP), use of mechanical ventilation, pressure support, vasoactive drug, SOFA score, systolic blood pressure (SBP), diastolic blood pressure (DBP), proteinuria, hematuria, lactate, need for dialysis (indication, type and duration).

All patients were followed up to resolution, discharge or death.

### Ethical Issues

This study was conducted in accordance with the basic ethical principles of the guidelines and regulatory norms for research involving human subjects as stated on Resolution 196/96. Approval from the National Research Committee (CONEP) was obtained on May 3, 2020 (CAEE 30451520.6.0000.5411 and review number: 4,003,880). Patients were informed about the study protocol, content and relevance, and enrolled by signing an informed consent form.

### Statistical Analyses

All consecutive patients hospitalized with a diagnosis of COVID-19 were divided into two subgroups: elderly patients who did not develop AKI during hospitalization and elderly in-patients who did develop AKI.

As per study protocol, data were entered, checked for possible typing errors and analyzed with the aid of the statistical software STATA 8.0 (Stata Corp, 2004). Data were expressed as mean and standard deviation or median and interquartile range. Significance was set at 5% (p<0.05).

First, a descriptive analysis of all elderly patients followed during the study period was conducted including measures of central tendency and dispersion for continuous variables, and frequency measures for categorical variables. The occurrence of AKI was then set as the dependent variable, and comparisons were performed using the Chi-Square test for categorical variables and the t-test or Mann-Whitney test for continuous variables.

Finally, multiple logistic regression was used to analyze the associations of all independent variables considered significant on univariate analysis (p ≤ 0,20) with the study outcome. Similar procedures were carried out using death occurrence as a dependent variable.

## Results

Throughout 2020, 347 patients with a confirmed diagnosis of COVID-19 were admitted to the Hospital of Clinics of Botucatu Medical School (HC-FMB UNESP). The highest rates of hospital admission were observed in July (17.9%), August (16.7%) and December (14.4%), with 52.4% of patients requiring intensive care unit (ICU) care. Patient mean age was 59.8 ± 16.1 years, with a predominance of males (57.6%). Mean length of stay was 12.6 ± 10.1 days.

**Figure 1:**
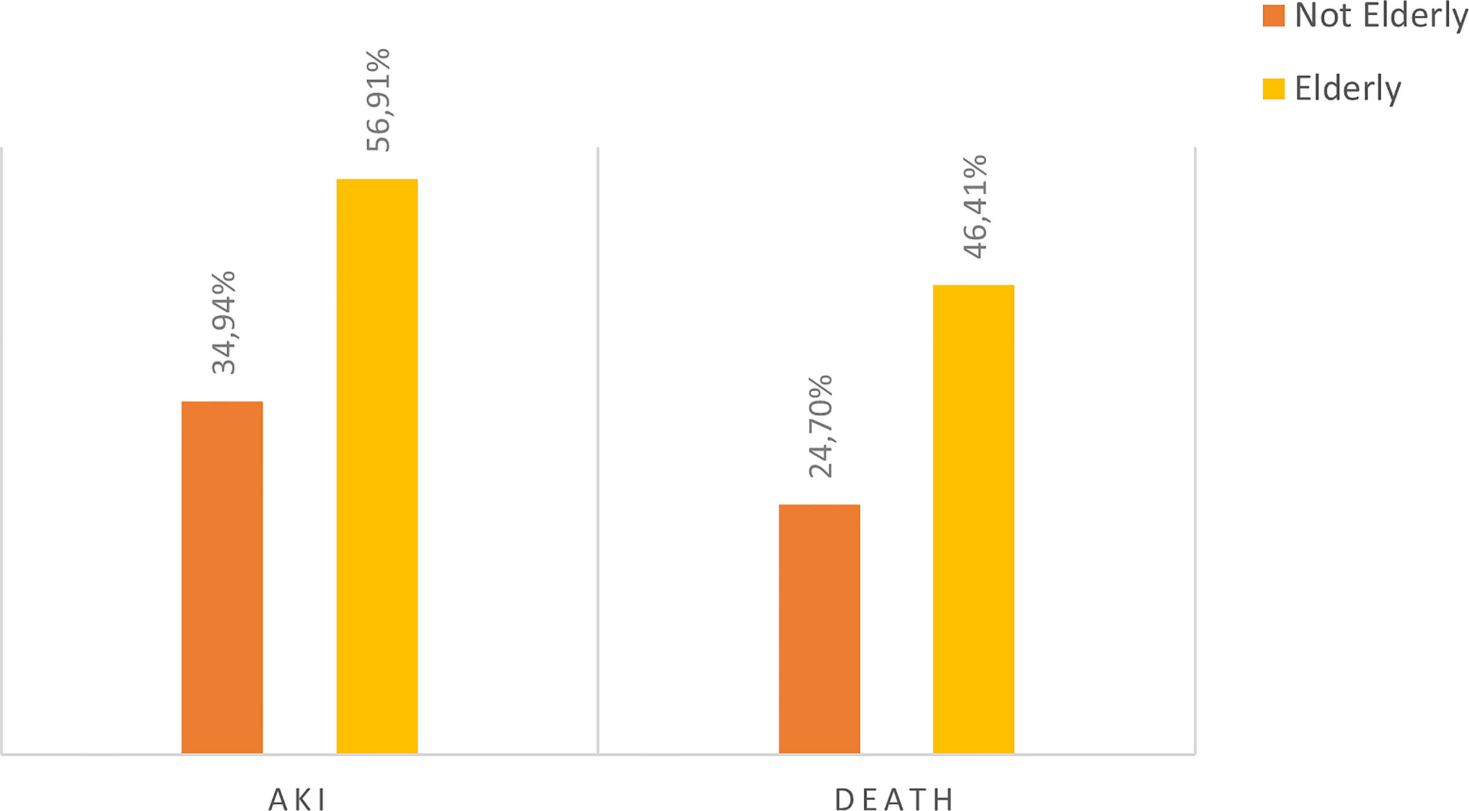
Distribution of the not elderly and elderly population with COVID-19 hospitalized at HC-FMB.

**Figure 2:**
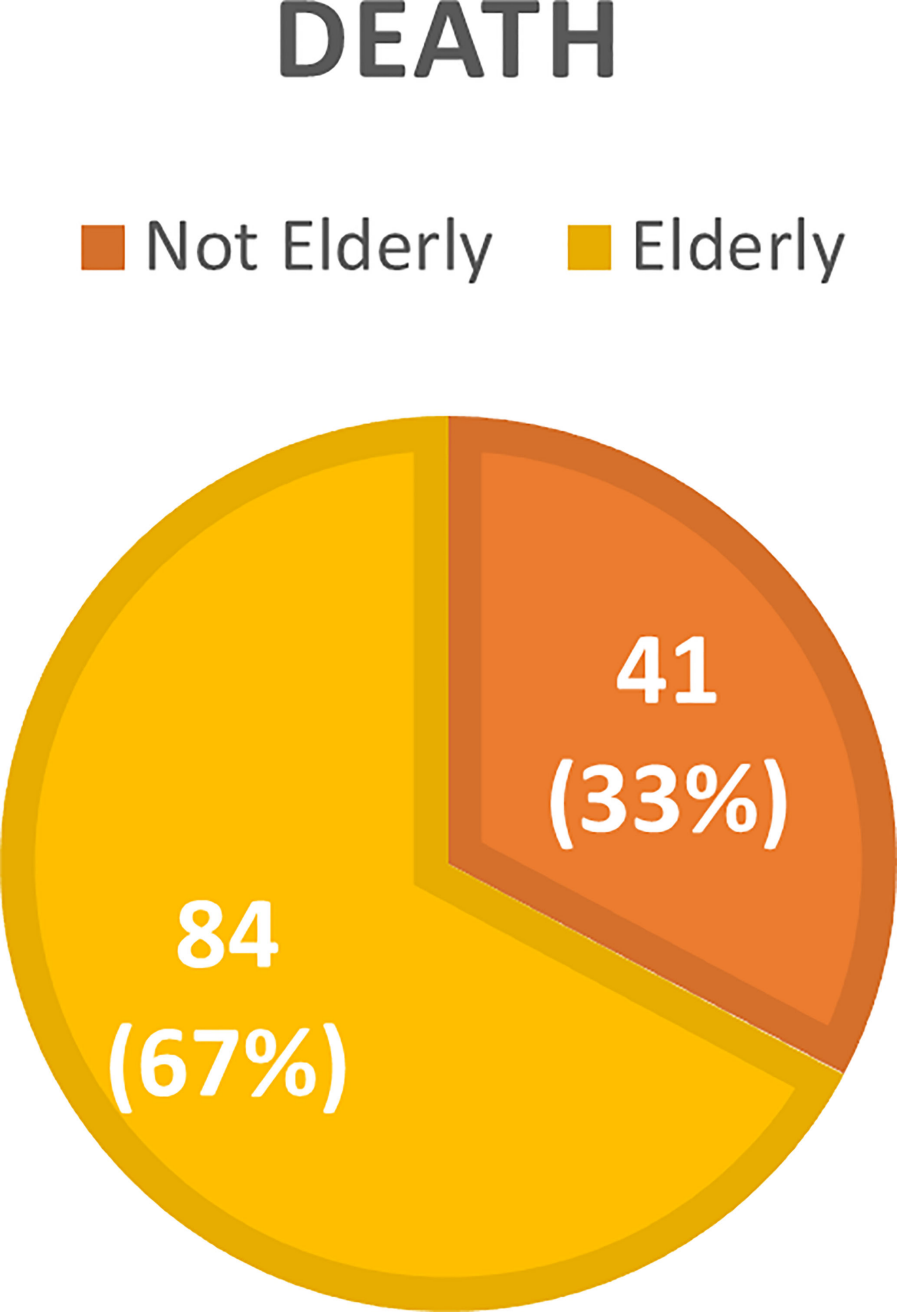
Distribution of the not elderly and elderly population with COVID-19 according to the presence or absence of Acute Kidney Injury (AKI).

**Figure 3:**
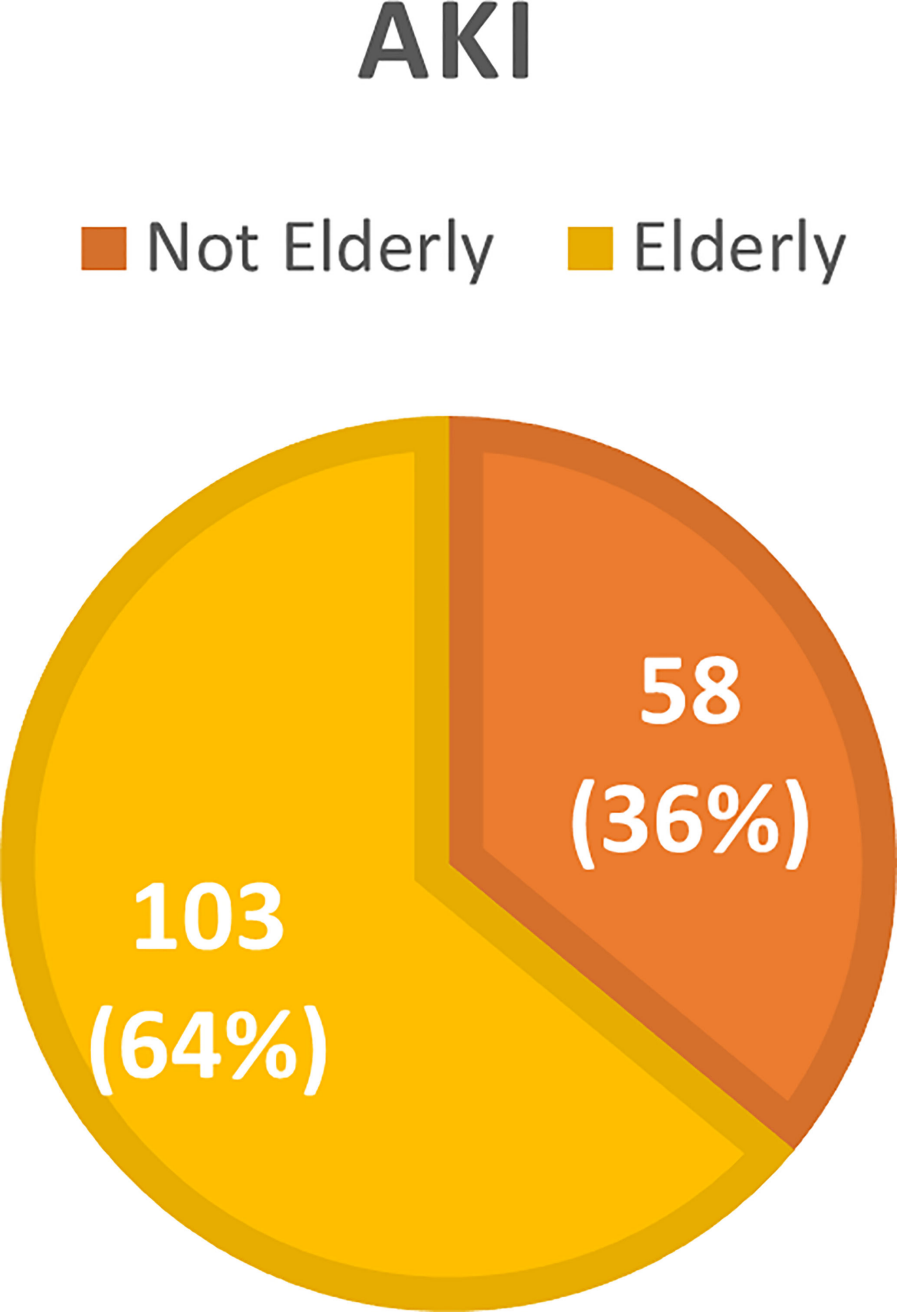
Distribution of the not elderly and elderly population with COVID-19 according to the evolution to death.

**Figure 4:**
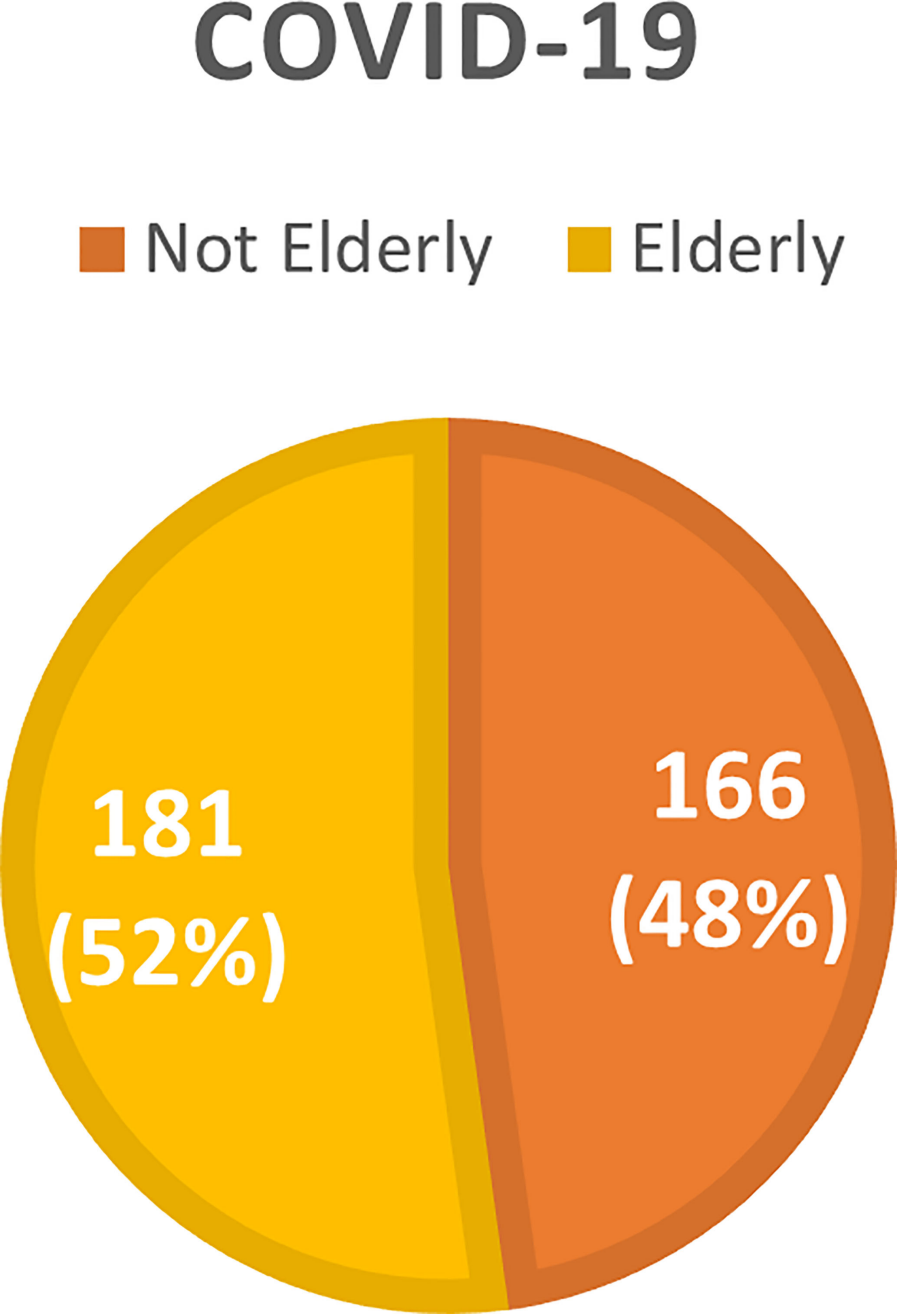
Distribution of the not elderly and elderly population with COVID-19 hospitalizes at HC-FMB according to the presence of AKI and the evolution to death.

The overall incidence of AKI was 46.4% with a predominance of KDIGO 3 (53.4%). Mean time to AKI diagnosis was 6 days. Acute kidney replacement therapy (AKRT) was required by 46.6%. Elderly patients accounted for 63.97% of the 161 patients who developed AKI, and 67.20% of the 125 individuals who died (**Figures 1**–**4**).

Of the 347 patients with COVID-19 admitted to our hospital during the study period, 181 (52.16%) were elderly, with a median age of 72 years (65- 80 years). In this age group, there was a predominance of males (56.91%), and most of them were hypertensive (73.48%). Moreover, 55.25% of them required ICU care, while 44.75% were admitted to the hospital ward. Of 129 elderly patients who underwent urinalysis at hospital admission, 46.51% and 57.36% had proteinuria and hematuria, respectively.

Among the elderly, AKI overall incidence was 56.9%, with a predominance of KDIGO 3 (50.48%), while incidence of KDIGO stage 1 and 2 was 25.24% and 22.33%, respectively. AKI incidence was higher in ICU patients (73.27%) as compared to those admitted to the ward (33.33%) (p < 0.001). Mean length of stay was longer (13.8 vs.19.75 days) for the elderly who developed AKI than for patients who did not (9, 5 vs. 15 days). AKRT was required by 49 (47.57%) of the elderly patients with AKI. Mean duration of AKRT was 6 ± 2 days. Death was also more frequent among patients with AKI than in those without (66.33% vs. 31.79%, p < 0.0001). Among elderly patients treated with AKRT, the mortality was very high (82%). The most elderly AKI patients surviving AKRT recovered partially from AKI after 30 days of discharge hospital (66.7%), while only 3(33.3%) had total recorvry of kidney function.

The factors associated with AKI development in the population over 60 years of age were the male sex (47.40% vs 64.08%, p = 0.037), higher baseline creatinine level (0. 60-0.90 vs 0.70-1.25, p = 0.007), higher creatine phosphokinase (CPK) (37.50-97.00 vs 75.00-544.75, p < 0.001), D-Dimer (312-2185 vs 4425-15640, p < 0.001), need for mechanical ventilation (10.25% vs 70.87%, p < 0.0001), use of vasoactive drugs (14.10% vs 68.93%, p < 0.0001), length of stay (5-15 vs 8-19.75, p < 0.001), need for ICU (33.33% vs 73.26%, p < 0.0001), APACHE score (9.00-20.00 vs 20.25-26.75, p = 0.009), presence of urinalysis proteinuria (29.41% vs 57.69%, p = 0.002) and hematuria (39.21% vs 69.23%, p < 0.001) (**Table 1**). Logistic regression confirmed higher baseline creatinine level (OR 10.54, CI 1.22 -90.61, p = 0.032) and need for mechanical ventilation (OR 9.26, CI 1.08-79.26, p = 0.042) as risk factors for the development of AKI (**Table 2**).

**Table 1:** Clinical and laboratory characteristics of elderly patients hospitalized with COVID-19 regarding the presence or absence of AKI.

Variables	General (n = 181)	Without AKI (n = 78)	With AKI (n = 103)	P Value
Male sex (%)	103 (56,91)	37 (47,44)	66 (64,08)	0,037
Age	72 (65-78)	72 (65-80)	72 (65,2-77,5)	0,94
ACE inhibitors use (%)	95 (52,49)	43 (55,13)	52 (52)	0,79
Arterial hypertension (%)	133 (73,48)	58 (74,36)	75 (75)	0,94
Diabetes (%)	83 (45,86)	35 (44,87)	48 (46,60)	0,79
Obesity (%)	38 (20,99)	16 (20,51)	22 (21,78)	0,99
CKD (%)	29 (16,02)	9 (11,54)	20 (19,42)	0,22
Cardiovascular disease (%)	48 (26,52)	20 (25,64)	28 (27,18)	0,95
Diuretic use (%)	62 (34,25)	27 (34,62)	35 (33,98)	0,94
Basal creatinine	0,8 (0,625-1)	0,8 (0,6-0,9)	0,85 (0,7-1,25)	0,007
CPK	98 (48-357)	66 (37,5-97)	288 (75-544,75)	<0,001
D dimer	5098 (1899-13296)	1126 (312-2185)	9789,5 (4425-15640)	<0,001
Proteinuria (%)	60 (46,51)	15 (29,41)	45 (57,69)	0,002
Hematuria (%)	74 (57,36)	20 (39,21)	54 (69,23)	<0,001
Mechanical ventilation (%)	79 (43,65)	8 (10,26)	71 (70,87)	<0,0001
Vasoactive drugs (%)	80 (44,20)	11 (14,10)	69 (68,93)	<0,001
ICU admission (%)	100 (55,25)	26 (33,33)	74 (73,27)	<0,0001
Length of stay	11 (7-15)	9 (5-15)	13 (8-19,75)	<0,001
APACHE	24 (20-25)	18 (9-20)	25 (20,25-26,75)	0,009
Death (%)	84 (46,41)	17 (31,79)	67 (66,34)	<0,001

AKI, acute kidney injury; ACE, angiotensin converting enzyme; CKD, chronic kidney disease; ICU, intensive care unit.

Obs. The values of n to Proteinuria and Hematuria were, respectively, equal to 51 and 78.

**Table 2:** Logistic regression for AKI.

VARIABLES	OR	CI	P VALUE
Basal creatinine	10,54	1,22-90,61	0,032
Mechanical ventilation	9,26	1,08-79,26	0,042

Overall mortality in the elderly population was 46.40%, with a higher incidence in patients requiring ICU care (29.47% vs 85.71%, p < 0.0001). Factors associated with mortality were higher baseline creatinine level (0.60-1.00 vs 0.72-1.10, p = 0.046), CPK (37-98 vs 76-565, p < 0.0001) and D-Dimer (1125.50-5234.25 vs 4535.25-15544.00, p < 0.0001), ICU admission (29.47% vs 85.71%, p < 0.0001), need for mechanical ventilation (10.53% vs 82.14%, p < 0.0001), use of vasoactive drugs (12.63% vs 80.95%, p < 0.0001), APACHE score (8.25-23, 00 vs 22.00-27.00, p = 0.0043) and SOFA score (3-9 vs 9-13, p = 0.001), AKI development (35.79% vs 79.76%, p < 0, 0001), KDIGO 3 (10.53% vs 48.81%, p = 0.0056), urinalysis at hospital admission showing hematuria (30.64% vs 81.53%, p < 0.0001) and proteinuria (32.25% vs 60.00%, p = 0.0031) (**Table 3**). On logistic regression analysis, higher CPK level (OR 1.009, CI 1.001-1.017, p = 0.042), need for mechanical ventilation (OR 17.71, CI 1.13-277.62, p = 0.002) and KDIGO 3 (OR 2.017 CI 1.039 -3.917, p = 0.038) remained associated with mortality (**Table 4**).

**Table 3:** Clinical and laboratory characteristics of elderly patients hospitalized with COVID-19 in terms of mortality.

Variables	No Death (n = 95)	Death (n = 84)	P Value
Male sex (%)	49 (51,58)	52 (61,90)	0,215
Age	71 (65-77)	73 (67-79)	0,085
Arterial hypertension (%)	71 (74,74)	62 (73,81)	0,867
Diabetes (%)	43 (45,26)	40 (47,62)	0,810
Obesity (%)	18 (18,95)	20 (23,81)	0,513
CKD (%)	15 (15,79)	14 (16,67)	0,964
Cardiovascular disease (%)	24 (25,26)	24 (28,57)	0,705
Basal creatinine	0,8 (0,6-1)	0,9 (0,72-1,1)	0,046
CPK	74 (37-98)	292 (76-565)	<0,0001
D dimer	1999 (1125-5234,25)	9215 (4535,25-15544)	<0,001
Proteinuria (%)	20 (32,25)	39 (60)	0,0031
Hematuria (%)	19 (30,64)	53 (81,53)	<0,0001
Mechanical ventilation (%)	10 (10,53)	69 (82,14)	<0,0001
Vasoactive drugs (%)	12 (12,63)	68 (80,95)	<0,0001
ICU admission (%)	28 (29,47)	72 (85,71)	<0,0001
AKI (%)	34 (35,79)	67 (79,76)	<0,0001
KDIGO 3 (%)	10 (10,53)	41 (48,81)	0,0056
APACHE	20 (8,25-23)	25 (22-27)	0,0043
SOFA	7 (3-9)	11 (9-13)	0,001

AKI, acute kidney injury; CKD, chronic kidney disease; ICU, intensive care unit.

Obs. The values of n to Proteinuria and Hematuria were, respectively, equal to 62 and 65.

**Table 4:** Logistic regression for death.

VARIABLES	OR	CI	P VALUE
CPK	1,009	1,001-1,017	0,042
Mechanical ventilation	17,71	1,13-277,62	0,002
KDIGO 3	2,017	1,039-3,917	0,038

## Discussion

This study describes the first wave of the COVID-19 pandemic in Brazil –from March to December 2020 – in a Public Tertiary Referral Hospital that provides care to patients referred from 68 municipalities outside the capital of São Paulo State (14). During the study period, data on 347 hospitalized patients diagnosed with COVID-19 were analyzed. The majority of patients were elderly (52.16%), with a mean age of 72 years, and under ICU care (54.94%). Moreover, the elderly accounted for 63.97% of the 161 patients who developed AKI, and 67.20% of the 125 cases of death.

Our results showed that AKI incidence was higher in older patients than in younger patients (56.91% vs 34.94%, p < 0.0001), and that the risk factors associated with AKI in the elderly were the male sex, higher levels of baseline creatinine, CPK and D-Dimer, need for mechanical ventilation, use of vasoactive drugs, longer length of hospital stay, ICU admission, higher APACHE score, and urinalysis at hospital admission indicating proteinuria and hematuria. On logistic regression, higher baseline creatinine and need for mechanical ventilation remained associated with AKI development.

In general, our findings corroborate Chinese, European and North American studies indicating that AKI in the new coronavirus infection occurs more frequently in men with advanced age showing comorbidities, such as obesity, hypertension, diabetes mellitus and chronic kidney disease (8, 13, 15). In China, the reported incidence of AKI in patients infected with SARS-CoV-2 is lower than that reported by Western studies (15, 16), ranging from 0.5% to 50% (15). This can be explained by the greater expression of the ACE-2 and TMPRSS genes – both necessary for the virus to invade host cells (2, 11, 12) – seen in Western individuals (11). In contrast, Zamoner et al. demonstrated that AKI frequency in Brazilian patients was higher than that reported in other European and North American studies that reach around 50% of in-patients with COVID-19 (9).

The physiopathology of AKI in COVID-19 is multifactorial. According to Menez and Parikh (2021), SARS-CoV-2 effects on kidney tissues may be direct or indirect (17). Direct viral effects include direct endothelial damage from viral entry, local inflammation, and collapsing glomerulopathy; while indirect effects include sepsis, nephrotoxic medications, and systemic inflammation, also known as cytokine storm (17).

The entry of the coronavirus into host cells is highly dependent on the co-expression of ACE-2 and TMPRSS (2, 11, 12). This co-expression is frequently found in podocytes and proximal tubule cells – which play a critical role in kidney function, and explains why patients infected with the virus tend to show proteinuria and hematuria on urinalysis (2, 9–13, 16), as well as in other tissues targeted by the virus, such as the lungs, heart, vessels, and liver, among others (10, 11, 16). Furthermore, histopathological and immunohistochemical findings in patients with COVID-19 associated with AKI indicate that this virus can cause severe necrosis in the kidney tubule cells besides promoting lymphocyte infiltration into the kidney tissue (15, 18).

Another mechanism that may also be involved in the development of AKI in COVID-19 is the storm of pro-inflammatory cytokines (IFN-γ, IL-6, IL-8 and TNF-α) induced by SARS-CoV-2, which activates nuclear factor kappa B (NF-kB) (2, 12, 13, 16, 18). Cytokine storm is known to lead to failure of multi-organs, including the kidneys (2, 12, 13, 16, 18). In addition, kidney injury itself plays a role in the cytokine storm produced by COVID-19 by hyperactivating the immune system to contribute to the processes of fibrosis, apoptosis and changes in the microvasculature. Hence, AKI has been associated with worse prognosis including multi-organ failure and death (12, 13).

Despite the knowledge so far accumulated, there are few studies addressing the development of AKI in elderly patients affected by COVID-19. This is a matter of great importance because aging itself has been indicated as a risk factor for worse diagnoses, as immunosenescence and the continuous production of pro-inflammatory mediators - typical of senescence – greatly contribute to SARS-CoV- 2 infection and the cytokine storm caused by the virus (6, 7).

As for mortality, an outcome associated with the development of AKI during infection, this study showed a higher death prevalence in elderly patients than in non-elderly individuals (46.41% vs 24.70%, p < 0.0001). Upon univariate analysis, higher values of baseline creatinine, CPK and D-Dimer, as well as ICU admission, need for mechanical ventilation, use of vasoactive drugs, higher APACHE and SOFA scores, development of AKI, KDIGO 3 AKI, and urinalysis showing proteinuria and hematuria were identified as risk factors for mortality in the elderly population. Logistic regression confirmed the association of higher CPK levels, need for mechanical ventilation and the presence of severe AKI (KDIGO 3) with death among the elderly.

This study presents some limitations as the number of elderly patients was not big and the data were obtained in a single center. Despite these limitations, this was the first study of the Latin America to determine AKI incidence and the risk factors associated with its development and outcome in elderly patients with COVID-19.

## Conclusion

In brief, this study showed that among the elderly patients with COVID-19, admitted to a public tertiary referral hospital located in the state of São Paulo, AKI development occurred more frequently in individuals of the male sex with a mean age of 72 years, and under ICU care. The risk factors associated with AKI development were need for mechanic ventilation and higher baseline creatinine. There was a predominance of AKI KDIGO 3, which contributed to a higher mortality, and highlights its importance as a predictor of adverse outcomes. In addition to severe AKI, higher CPK levels and need for mechanic ventilation were also identified as risk factors associated with death.

Further studies on the association of AKI development and death in elderly patients with COVID-19 are warranted.

## Data Availability Statement

The original contributions presented in the study are included in the article/supplementary materials. Further inquiries can be directed to the corresponding authors.

## Ethics Statement

This study was reviewed and approved by the National Research Committee (CONEP), obtained on May 3, 2020 (CAEE 30451520.6.0000.5411 and review number: 4,003,880). The patients/participants provided their written informed consent to participate in this study.

## Author Contributions

Concept/design, DP and BY. Data analysis/interpretation, DP, BY, LM, and PS. Drafting article, DP and BY. Critical revision of article, DP, AB, and WZ. Approval of article, DP and BY. Statistics, DP and WZ. Funding secured by BY and DP. Data collection, BY and LM. All authors contributed to the article and approved the submitted version.

## Funding

The study is funded by “Conselho Nacional de Desenvolvimento Científico e Tecnológico” (CNPq 126177/2021-0).

## Conflict of Interest

The authors declare that the research was conducted in the absence of any commercial or financial relationships that could be construed as a potential conflict of interest.

## Publisher’s Note

All claims expressed in this article are solely those of the authors and do not necessarily represent those of their affiliated organizations, or those of the publisher, the editors and the reviewers. Any product that may be evaluated in this article, or claim that may be made by its manufacturer, is not guaranteed or endorsed by the publisher.
